# Development and Validation of a Scoring System (SAGA Score) to Predict Weight Loss in Community-Dwelling, Self-Supported Older Adults

**DOI:** 10.3390/nu16121848

**Published:** 2024-06-13

**Authors:** Eiji Sadashima, Hirokazu Takahashi, Yoshitaka Koga, Keizo Anzai

**Affiliations:** 1 Medical Research Institute, Saga-Ken Medical Centre Koseikan, Saga 840-8571, Japan; 2 Division of Metabolism and Endocrinology, Faculty of Medicine, Saga University, Saga 849-8501, Japan; takahas2@cc.saga-u.ac.jp (H.T.); akeizo@cc.saga-u.ac.jp (K.A.); 3 Liver Center, Saga University Hospital, Faculty of Medicine, Saga University, Saga 849-8501, Japan; 4 Saga Prefectural Tosu Health and Welfare Office, Saga 841-0051, Japan; koga-yoshitaka@pref.saga.lg.jp

**Keywords:** weight loss, community-dwelling, self-supported older adults, scoring system, Kokuho Database system

## Abstract

This retrospective cohort study explored the prevalence of substantial weight loss (≥10% per year) in independent older individuals in order to develop and validate a scoring system for high-risk group identification and targeted intervention against malnutrition. We used insurance claims and the Kokuho Database (KDB), a nationwide repository of Japanese-specific health checkups and health assessments for the older people. The study included 12,882 community-dwelling individuals aged 75 years and older who were self-supported in their activities of daily living in Saga Prefecture, Japan. Health evaluations and questionnaires categorized weight-loss factors into organic, physiological, psychological, and non-medical domains. The resulting scoring system (SAGA score), incorporating logistic regression models, predicted ≥ 10% annual weight-loss risk. The results revealed a 1.7% rate of annual substantial weight loss, with the SAGA score effectively stratifying the participants into low-, intermediate-, and high-risk categories. The high-risk category exhibited a weight-loss rate of 17.6%, highlighting the utility of this scoring system for targeted prevention. In conclusion, the validated SAGA score is a crucial tool for identifying individuals at high risk of significant weight loss, enabling tailored interventions and social support benefiting both older individuals and their relatives.

This retrospective cohort study explored the prevalence of substantial weight loss (≥10% per year) in independent older individuals in order to develop and validate a scoring system for high-risk group identification and targeted intervention against malnutrition. We used insurance claims and the Kokuho Database (KDB), a nationwide repository of Japanese-specific health checkups and health assessments for the older people. The study included 12,882 community-dwelling individuals aged 75 years and older who were self-supported in their activities of daily living in Saga Prefecture, Japan. Health evaluations and questionnaires categorized weight-loss factors into organic, physiological, psychological, and non-medical domains. The resulting scoring system (SAGA score), incorporating logistic regression models, predicted ≥ 10% annual weight-loss risk. The results revealed a 1.7% rate of annual substantial weight loss, with the SAGA score effectively stratifying the participants into low-, intermediate-, and high-risk categories. The high-risk category exhibited a weight-loss rate of 17.6%, highlighting the utility of this scoring system for targeted prevention. In conclusion, the validated SAGA score is a crucial tool for identifying individuals at high risk of significant weight loss, enabling tailored interventions and social support benefiting both older individuals and their relatives.

## 1. Introduction

As the older population continues to increase globally, optimal nutritional status should be maintained among community-dwelling, self-supported members. Weight loss is a common predictor of nutritional status. Several epidemiological studies have reported an association between weight loss and increased mortality [1,2,3,4,5]. A Cochrane review of randomized and quasi-randomized controlled trials indicated that providing extra energy and protein to undernourished older individuals leads to weight gain and decreased mortality. This review substantiated the causal relationship between undernutrition and mortality, highlighting the positive impact of nutritional interventions in this population [6,7].

In Japan, promoting good nutrition and addressing malnutrition among older adults are important health goals of the National Health Promotion Movement for the 21st Century, known as Healthy Japan 21. This initiative aims to extend healthy life expectancy. Currently, various healthcare programs, including health checkups, are conducted for individuals aged > 75 years or those aged 65–74 years with disability certification under the programs’ criteria. These programs were designed to evaluate the health and nutritional status of older adults. The results indicate that nutritional and health guidance must be provided efficiently according to individual risk levels. To meet this need, the Wide-Area Union for the Medical Care System for the Elderly has been tasked with enhancing healthcare programs for frailty since fiscal year (FY) 2020.

However, the prevailing perception is that most community-dwelling, self-supported older people maintain their weight [1,8,9,10], and currently, there is no explicit criterion for intensive guidance. One approach is to use prediction models to quantify the risk of adverse outcomes. This enables the identification of high-risk individuals.

This study aimed to develop and validate a scoring system capable of predicting weight loss in a community-dwelling, self-supported older population. Such a scoring system would enable healthcare providers to identify individuals at a heightened risk of weight loss, facilitating the implementation of appropriate interventions to prevent malnutrition and the associated adverse health outcomes.

## 2. Materials and Methods

### 2.1. Study Samples

**Figure 1:**
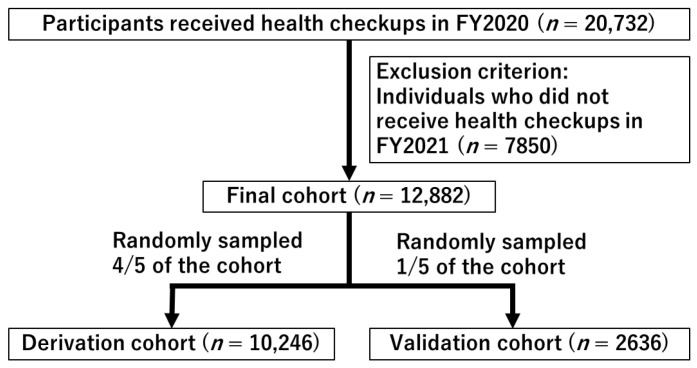
Flow chart illustrating the enrollment of participants for the cohort study, detailing the inclusion and exclusion criteria and the final enrollment numbers.

This study leveraged health and long-term care insurance claims, as well as the Kokuho Database (KDB), a nationwide repository encompassing Japanese-specific health checkup and health assessment data of older adults. A retrospective cohort design was employed, involving 20,732 older individuals who underwent health checkups in FY2020. The participants, aged 75 years and older, resided in Saga Prefecture, Japan. The participants were non-institutionalized, community-dwelling individuals who were self-supported in their activities of daily living. The estimated coverage of these health checkups represents 24.3% of the community-dwelling, self-supported older population in the prefecture. A total of 7850 participants without health checkups in FY2021 were excluded, resulting in a final enrollment of 12,882 participants in this study (Figure 1). Information about individuals who did not receive health checkups cannot be obtained from KDB. The Medical Ethics Committee of Saga Medical Centre Koseikan reviewed and approved the study design (permission number: 22-08-01-01).

### 2.2. Variables and Outcome

Health checkups for the older individuals included physical examinations, blood screening tests, and standardized questionnaires. Various health conditions, such as hypertension, diabetes mellitus (DM), dyslipidemia, hyperuricemia, liver function abnormalities, chronic kidney disease (CKD), chronic obstructive pulmonary disease (COPD), pneumonia, cardiovascular diseases, musculoskeletal diseases, cancer, dementia, depression, and schizophrenia were identified based on the medical history or health checkup criteria for the older individuals. A standardized questionnaire was administered to collect information on weight loss, smoking status, alcohol consumption, dietary intake, cognitive function, and physical activity (Table 1). The percent of weight loss among the 12,882 participants who underwent health checkups in FY2021 was calculated as follows:

(1) $$Percent\;weight\;loss=\frac{\left(Weight\;in\;FY2020-Weight\;in\;FY2021\right)}{Weight\;in\;FY2020}\times 100$$

The weight-loss rate per year was calculated based on the date of measurement for each participant. The outcome was defined as weight loss ≥ 10% per year according to the European Society of Clinical Nutrition and Metabolism (ESPEN) guidelines [11].

### 2.3. Statistical Analysis

All participants were divided into derivation and validation cohorts in a 4:1 ratio using a random numbers table (Figure 1). The distribution between the two groups were compared using Pearson’s chi-square test or Fisher’s exact test. Standardized differences between the derivation and validation cohorts were calculated to assess the balance. A scoring system was developed using baseline characteristic data to predict weight loss ≥ 10% per year. Logistic regression models were used to compute crude odds ratios (ORs), adjusted ORs, and 95% confidence intervals (CIs) for weight loss ≥ 10% per year. Model selection for multivariable analyses followed the stepwise method, incorporating all factors with *p* values < 0.10 on univariate analyses. The final model, identified based on the Akaike information criterion, yielded the predictor weights calculated from the model coefficients. Each rounded coefficient was assigned a corresponding value using the scoring system.

The discriminatory power of the scores was assessed using the area under the receiver operating characteristic curve (ROC-AUC). The scores were categorized into tertiles (low-, intermediate-, and high-risk categories), and the Cochran–Armitage trend test was used to test the statistical significance of the observed proportions of events across risk categories. A validation cohort was used to test the validity of the prediction scores. Calibration was performed using a visual calibration plot to compare the actual and predicted probabilities of events [12].

For missing values, a complete case analysis was conducted if the missing values were <1%, which was deemed feasible. The study adhered to the TRIPOD checklist of prediction models [13]. All analyses were conducted using R software (version 4.2.2, https://www.r-project.org (accessed on 31 October 2022)), and significance was set at *p* < 0.05.

## 3. Results

### 3.1. Characteristics of the Participants

Table 2 presents the characteristics of the participants, with 10,246 individuals enrolled in the derivation cohort and 2636 in the validation cohort (Figure 1). There were no significant differences in participant characteristics between the two cohorts, except for question 4 (Q4) of the questionnaire (“Compared to 6 months ago, do you find it more difficult to eat tough or solid foods”). The standardized differences in all variables were less than 0.1, indicating a well-balanced distribution between the two cohorts. The incidence of weight loss ≥ 10% per year was 1.7% (176/10,246) in the derivation cohort and 1.7% (44/2636) in the validation cohort.

### 3.2. Development and Discrimination of the Scoring System in the Derivation Cohort

**Figure 2:**
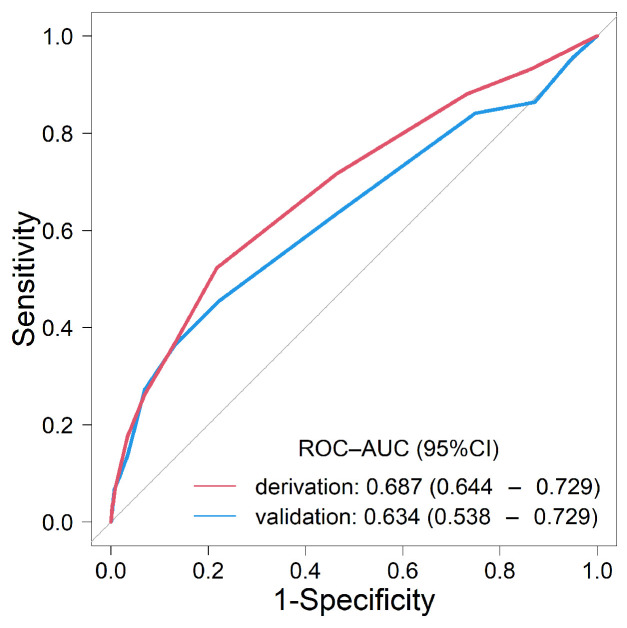
Receiver operator characteristic (ROC) curves demonstrating the predictive performance of the SAGA score for identifying weight loss of ≥10% per year in both the derivation and validation cohorts.

**Figure 3:**
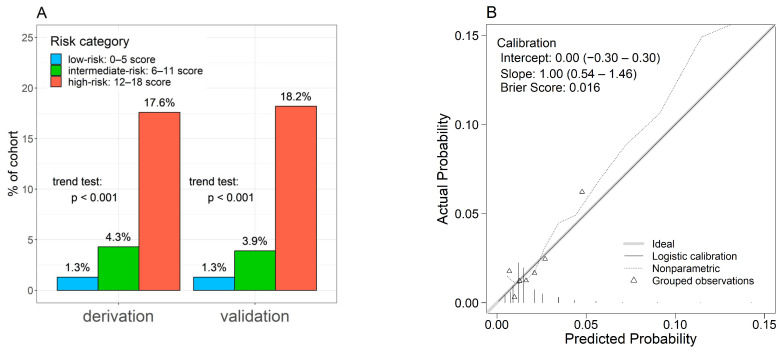
Predictive ability and calibration of the predictive score for a weight loss of ≥10% per year. (**A**) Rate of percent weight loss of ≥10% per year across risk categories defined by the SAGA score. (**B**) Calibration plot illustrating the agreement between predicted and actual probabilities of weight loss of ≥10% per year.

Univariate analyses revealed 22 factors as candidate predictors (*p* < 0.10) for weight loss ≥ 10% per year (Table 3). Multivariable analysis highlighted nine factors: age ≥ 90 years, male gender, albumin level < 3.5 g/dL, CKD, COPD, musculoskeletal disorders, dementia, depression, and a positive response to question 7 (Q7) of the questionnaire (“Do you think your walking speed has slowed down compared to before?”). A novel weighted score was developed using the coefficients from multivariable analysis (Table 4). The ROC-AUC (95% CI) of the score was 0.687 (0.644–0.729) (Figure 2). According to the scoring system, risk categories were defined as follows: scores of 0–5 were considered low risk, 6–11 as intermediate risk, and 12 or more as high risk. Weight loss of ≥ 10% per year in the low-, intermediate-, and high-risk categories was 1.3% (111/8848), 4.3% (59/1364), and 17.6% (6/34), respectively (Figure 3A: derivation cohort, *p* < 0.001 for trend).

### 3.3. Application of the Scoring System in the Validation Cohort

ROC analysis of the validation cohort yielded a ROC-AUC (95% CI) of 0.634 (0.538–0.729) (Figure 2). Weight loss of ≥10% per year in the low-, intermediate-, and high-risk categories was 1.3% (29/2205), 3.9% (13/330), and 18.2% (2/11), respectively (Figure 3A: validation cohort, *p* < 0.001 for trend). The calibration plot demonstrated a good fit between the predicted probabilities of weight loss ≥ 10% per year and the actual prevalence rates. The intercept (95% CI) was 0.00 (−0.3–0.3), the slope (95% CI) was 1.00 (0.54–1.46), and the Brier score was 0.016, suggesting good calibration (Figure 3B).

## 4. Discussion

In this cohort study, we revealed that the rate of weight loss ≥ 10% per year is 1.7% among the community-dwelling, self-supported population aged 75 years and older. These findings align with those from the study by Kobayashi et al. (*n* = 18,674), which reported a rate of 1.2% for weight loss ≥ 10% per year [14], suggesting consistency across studies. Therefore, effective intervention strategies are required for such patients. To address this, we developed a new scoring system for weight loss ≥ 10% per year to identify high-risk groups within a large population. The scoring system, called the SAGA score (Self-supported elderly people AGe-related weight loss Assessment score), demonstrated a discriminatory power of 0.687 based on the ROC-AUC, with the identified high-risk category showing a substantial rate of 17.6% for weight loss ≥ 10% per year. This high-risk category exhibited a higher rate than the other risk categories, indicating effective stratification of risk. These findings suggest that targeted nutrition and health guidance for high-risk individuals could prevent weight loss.

Various factors are associated with weight loss in community-dwelling older populations. These factors are classified as organic (e.g., neoplastic, non-neoplastic, and age-related changes), physiological (e.g., chronic and acute diseases), psychological (e.g., depression and dementia), and non-medical (e.g., isolation and social problems) [1,2,5,15]. We conducted a comprehensive evaluation of these factors through multivariable analysis, and the resulting SAGA score included predictive factors such as age ≥ 90 y.o, male gender, albumin level < 3.5 g/dL, CKD, COPD, musculoskeletal disorders, dementia, depression, and a positive response for Q7 of the questionnaire (“Walking slower than before”). These factors contribute to weight loss through mechanisms such as decreased dietary intake, increased energy expenditure, and decreased muscle function [1,2,5,16,17,18]. Among older populations in Europe and the USA, socioeconomic status is identified as a risk factor [1,2,5]. In Japan, socioeconomic disparities are low and are not currently included as survey items. However, due to recent changes in social conditions, it will be necessary to investigate socioeconomic factors in the future.

Notably, an albumin level < 3.5 g/dL is assigned five points, indicating a strong association with weight loss. Albumin, the most abundant plasma protein, is primarily produced in the liver and is a crucial marker of nutritional status. However, the decrease in albumin levels can be caused by various factors such as malnutrition, chronic disease, muscle weakness, cancer, and vascular trauma. Recent studies have reported limitations in nutritional evaluation using albumin [19,20]. By contrast, Kobayashi et al. suggested an association between weight loss and albumin levels, and our results are similar [14]. Given that food intake regulation becomes less appropriate with age, nutritional therapy and proactive approaches are recommended to maintain nutritional status and albumin levels, potentially leading to weight gain. Older populations living alone, especially males, tend to have reduced food intake and are at a significantly higher risk of malnutrition [2]. One of the treatment strategies for CKD includes reducing protein intake. The gradual decrease in dietary intake (energy and calories) can lead to malnutrition, highlighting the complexity of prescribing nutritional care for older adults with CKD [17,21]. About half of patients with COPD experience weight loss, ranging from mild to severe [16,22]. This weight loss may be associated with a reduction in both muscle and adipose tissue. Additionally, COPD increases the basal metabolic rate due to the extra work required for breathing and/or systemic inflammation. The combination of hypermetabolism and decreased appetite lead to weight loss [16,23]. Dementia, particularly Alzheimer’s type, is associated with anorexia and weight loss [2,18]. Eating disorders are one of the symptoms of dementia [2,18,24], and weight loss is an early manifestation [2,18,25]. Depression is one of the most common reversible causes of anorexia and weight loss in the older populations [2,26]. Additionally, it is often accompanied by the loss of social networks, grief, and a sense of loss. This deterioration in social networks may lead to anorexia through various social factors [2]. Musculoskeletal disorders and “walking slower than before” impair physical capabilities, affecting activities of daily living such as shopping, meal preparation, and eating, which can lead to weight loss [2].

The strengths of our study include the large sample size of the community-dwelling, self-supported older population aged 75 years and older utilizing the KDB system in Japan. The KDB is a standardized database that includes data on health checkups and insurance claims with few missing values. Therefore, a SAGA score with multiple risk factors could be developed. Additionally, the scoring system demonstrated robust performance across both the derivation and validation cohorts, supporting the generalizability of our findings to community-dwelling, self-supported older populations. However, this study has limitations, as it was a retrospective study, introducing the possibility of selection bias. In particular, the assessment of individuals who did not receive health checkups could not be performed. However, a comparison of baseline characteristics between those excluded from the final cohort (*n* = 7850) and the final cohort (*n* = 12,882) revealed higher incidences of age ≥ 90 y.o, albumin level < 3.5 g/dL, CKD, dementia, and a positive response for Q7 of the questionnaire (“Walking slower than before”) in those excluded from the final cohort (Supplementary Table S1). This is consistent with the high proportion of participants in the intermediate- or high-risk categories according to the SAGA score, suggesting that the excluded data may include many participants who did not receive health checkups due to worsened clinical development. Therefore, early intervention is necessary for participants who are in the intermediate- or high-risk categories. Additionally, the study participants were only from the Saga Prefecture, Japan, necessitating validation of the scoring system using data from other regions.

## 5. Conclusions

In conclusion, we successfully developed and validated a scoring system. The use of this system to identify individuals at high risk of weight loss ≥ 10% per year will enable focused nutritional and health guidance for individuals, accompanied by appropriate social support. This valuable information can benefit community-dwelling, self-supported older people and their relatives.

## Figures and Tables

**Table 1:** Questionnaire of health checkups for the older adults, detailing the questions included in the assessment.

No.	Questions	Choices
Q1	How would you describe your current health status?	① Good ② Somewhat good ③ Normal ④ Not very good ⑤ Not good
Q2	Are you satisfied with your daily life?	① Satisfied ② Somewhat Satisfied ③ Somewhat unsatisfied ④ Unsatisfied
Q3	Do you regularly eat three meals a day?	① Yes ② No
Q4	Compared to 6 months ago, do you find it more difficult to eat tough or solid foods?	① Yes ② No
Q5	Have you ever choked on tea or soup?	① Yes ② No
Q6	Have you lost 2–3 kg or more in the past 6 months?	① Yes ② No
Q7	Do you think your walking speed has slowed down compared to before?	① Yes ② No
Q8	Have you fallen down previously in the past year?	① Yes ② No
Q9	Do you engage in exercise such as walking at least once a week?	① Yes ② No
Q10	Have you been told by others that you are forgetful, such as “always asking the same thing”?	① Yes ② No
Q11	Do you sometimes have no idea what the date is today?	① Yes ② No
Q12	Do you smoke?	① Yes ② No ③ No, but I used to.
Q13	Do you go out at least once a week?	① Yes ② No
Q14	Do you usually meet with family or friends?	① Yes ② No
Q15	When you are not feeling well, do you have someone to talk to?	① Yes ② No

**Table 2:** Summary of participant characteristics in both the derivation and validation cohorts.

Factors	Derivation Cohort (*n* = 10,246)	Validation Cohort (*n* = 2636)	Standardized Difference	*p* Value
Distribution by age			0.016	0.764
75–79 y.o	4842 (47.3)	1266 (48.0)		
80–89 y.o	4823 (47.1)	1226 (46.5)		
90 y.o and older	581 (5.7)	144 (5.5)		
Male gender	4200 (41.0)	1065 (40.4)	0.012	0.594
BMI < 18.5	770 (7.5)	188 (7.1)	0.015	0.532
Albumin level < 3.5 g/dL	96 (0.9)	26 (1.0)	0.005	0.822
Health history				
Hypertension	7657 (74.7)	1995 (75.7)	0.022	0.326
Diabetes mellitus	3417 (33.3)	865 (32.8)	0.011	0.610
Dyslipidemia	6501 (63.4)	1711 (64.9)	0.030	0.166
Hyperuricemia	1323 (12.9)	334 (12.7)	0.007	0.769
Liver function abnormality	1201 (11.7)	299 (11.3)	0.012	0.610
Chronic kidney disease	1703 (16.6)	431 (16.4)	0.007	0.769
Chronic obstructive pulmonary disease	1078 (10.5)	301 (11.4)	0.029	0.191
Pneumonia	1377 (13.4)	349 (13.2)	0.006	0.822
Cardiovascular disease	5871 (57.3)	1525 57.9)	0.011	0.612
Musculoskeletal disorders	8104 (79.1)	2110 (80.0)	0.024	0.293
Cancer	1332 (13.0)	320 (12.1)	0.026	0.253
Dementia	578 (5.6)	139 (5.3)	0.016	0.505
Depression	563 (5.5)	130 (4.9)	0.025	0.266
Schizophrenia	119 (1.2)	25 (0.9)	0.021	0.406
Questionnaire of special health checkups				
Q1: ④ Not very good or ⑤ Not good	836 (8.2)	233 (8.9)	0.025	0.251
Q2: ③ Somewhat unsatisfied or ④ Unsatisfied	686 (6.7)	194 (7.4)	0.026	0.225
Q3: ① Yes	9846 (96.1)	2530 (96.0)	0.006	0.779
Q4: ① Yes	2700 (26.4)	764 (29.0)	0.059	0.007
Q5: ① Yes	2088 (20.4)	549 (20.8)	0.011	0.607
Q6: ① Yes	1202 (11.7)	317 (12.0)	0.009	0.685
Q7: ① Yes	5475 (53.4)	1445 (54.8)	0.028	0.212
Q8: ① Yes	1855 (18.1)	499 (18.9)	0.021	0.337
Q9: ① Yes	6454 (63.0)	1617 (61.3)	0.034	0.119
Q10: ① Yes	1487 (14.5)	382 (14.5)	0.001	1.000
Q11: ① Yes	2271 (22.2)	547 (20.8)	0.034	0.119
Q12: ① Yes	369 (3.6)	102 (3.9)	0.014	0.522
Q13: ① Yes	8642 (84.3)	2205 (83.6)	0.019	0.385
Q14: ① Yes	9820 (95.8)	2504 (95.0)	0.041	0.060
Q15: ① Yes	9864 (96.3)	2524 (95.8)	0.027	0.211
Outcome				
Weight loss ≥ 10% per year	176 (1.7)	44 (1.7)	0.004	0.933

Abbreviations: y.o, years old; BMI, body mass index.

**Table 3:** Results of univariate analysis assessing factors associated with a weight loss of ≥ 10% per year in the derivation cohort.

Factors	Crude OR (95% CI)	*p* Value
Age ≥ 90 y.o	3.111 (2.045–4.732)	<0.001
Male gender	1.498 (1.088–2.062)	0.013
BMI < 18.5	1.149 (0.673–1.962)	0.609
Albumin level < 3.5 g/dL	7.830 (4.101–14.951)	<0.001
Hypertension	1.474 (1.008–2.156)	0.046
Diabetes mellitus	0.982 (0.715–1.349)	0.911
Dyslipidemia	0.914 (0.673–1.241)	0.562
Hyperuricemia	1.066 (0.690–1.647)	0.773
Liver function abnormality	1.626 (1.099–2.406)	0.015
Chronic kidney disease	1.964 (1.407–2.741)	<0.001
Chronic obstructive pulmonary disease	1.917 (1.301–2.827)	0.001
Pneumonia	1.498 (1.022–2.195)	0.038
Cardiovascular disease	1.449 (1.059–1.983)	0.021
Musculoskeletal disorders	2.208 (1.369–3.562)	0.001
Cancer	1.217 (0.804–1.840)	0.352
Dementia	2.713 (1.749–4.208)	<0.001
Depression	2.933 (1.906–4.514)	<0.001
Schizophrenia	3.684 (1.692–8.021)	0.001
Questionnaire of specific health checkups		
Q1: ④ Not very good or ⑤ Not good	1.534 (0.968–2.432)	0.069
Q2: ③ Somewhat unsatisfied or Unsatisfied	1.110 (0.627–1.963)	0.721
Q3: ① Yes	1.154 (0.508–2.623)	0.733
Q4: ① Yes	1.311 (0.953–1.805)	0.098
Q5: ① Yes	1.391 (0.991–1.952)	0.057
Q6: ① Yes	1.496 (0.999–2.241)	0.050
Q7: ① Yes	1.939 (1.406–2.674)	<0.001
Q8: ① Yes	1.338 (0.936–1.911)	0.109
Q9: ① Yes	0.624 (0.463–0.840)	0.002
Q10: ① Yes	1.420 (0.973–2.074)	0.069
Q11: ① Yes	1.568 (1.135–2.167)	0.006
Q12: ① Yes	0.618 (0.228–1.676)	0.345
Q13: ① Yes	0.744 (0.512–1.082)	0.120
Q14: ① Yes	1.048 (0.489–2.247)	0.904
Q15: ① Yes	1.677 (0.620–4.539)	0.309

Abbreviations: y.o, years old; BMI, body mass index; OR, odds ratio; CI, confidence interval.

**Table 4:** Results of multivariable analysis assessing factors associated with a weight loss of ≥10% per year in the derivation cohort.

Factors	Adjusted OR (95% CI)	*p* Value	Coefficient	Score
Age ≥ 90 y.o	1.923 (1.221–3.030)	0.005	0.654	2
Male gender	1.365 (0.982–1.897)	0.065	0.311	1
Albumin level < 3.5 g/dL	4.504 (2.259–8.979)	<0.001	1.505	5
Chronic kidney disease	1.579 (1.116–2.234)	0.010	0.457	1
Chronic obstructive pulmonary disease	1.664 (1.115–2.481)	0.012	0.509	2
Musculoskeletal disorders	1.631 (0.999–2.662)	0.050	0.489	2
Dementia	1.613 (1.004–2.592)	0.048	0.478	2
Depression	2.333 (1.492–3.647)	<0.001	0.847	3
Q7: Do you think your walking speed has slowed down compared to before? ① Yes	1.547 (1.113–2.150)	0.009	0.436	1

Abbreviations: y.o, years old; OR, odds ratio; CI, confidence interval.

## Data Availability

The original contributions presented in the study are included in the article, and further inquiries can be directed to the corresponding authors.

## Supplementary Materials

The following supporting information can be downloaded at: https://www.mdpi.com/article/10.3390/nu16121848/s1, Table S1: Summary of subject characteristics in the final cohort and excluded from final cohort.
