# Supplementary material for: Development and Validation of a Scoring System (SAGA Score) to Predict Weight Loss in Community-Dwelling, Self-Supported Older Adults
Source: Nutrients. 2024 Jun 13;16(12):1848. doi: 10.3390/nu16121848 (PMC11206483; doi:10.3390/nu16121848)
Supplement: Supplementary file 1 [file nutrients-16-01848-s001.zip › nutrients-3039264-supplementary.pdf]

**Supplementary Table S1.** Summary of subject characteristics in the final cohort and excluded from final cohort.

| Factors | Final cohort (*n* = 12,882) | Excluded from the final cohort (*n* = 7850) | Standardized difference | *p* value |
|---|---|---|---|---|
| Distribution by age | | | 0.239 | < 0.001 |
| 75–79 y.o | 6108 (47.4) | 2952 (37.6) | | |
| 80–89 y.o | 6049 (47.0) | 4080 (52.0) | | |
| 90 y.o and older | 725 (5.6) | 818 (10.4) | | |
| Male gender | 5265 (59.1) | 3093 (60.6) | 0.030 | 0.037 |
| BMI < 18.5 | 958 (7.4) | 723 (9.2) | 0.064 | < 0.001 |
| Albumin level < 3.5 g/dl | 122 (0.9) | 219 (2.8) | 0.136 | < 0.001 |
| Health history | | | | |
| Hypertension | 9652 (74.9) | 6139 (78.2) | 0.077 | < 0.001 |
| Diabetes mellitus | 4282 (33.2) | 2812 (35.8) | 0.054 | < 0.001 |
| Dyslipidemia | 212 (63.7) | 4934 (62.9) | 0.019 | 0.196 |
| Hyperuricemia | 1657 (12.9) | 1087 (13.8) | 0.029 | 0.045 |
| Liver function abnormality | 1500 (11.6) | 981 (12.5) | 0.026 | 0.067 |
| Chronic kidney disease | 2134 (16.6) | 1769 (22.5) | 0.151 | < 0.001 |
| Chronic obstructive pulmonary disease | 1379 (10.7) | 1021 (13.0) | 0.071 | < 0.001 |
| Pneumonia | 1726 (13.4) | 1305 (16.6) | 0.090 | < 0.001 |
| Cardiovascular disease | 7396 (57.4) | 5009 (63.8) | 0.131 | < 0.001 |
| Musculoskeletal disorders | 10214 (79.3) | 6403 (81.6) | 0.057 | < 0.001 |
| Cancer | 1652 (12.8) | 1198 (15.3) | 0.070 | < 0.001 |
| Dementia | 717 (5.6) | 929 (11.8) | 0.224 | < 0.001 |
| Depression | 693 (5.4) | 573 (7.3) | 0.079 | < 0.001 |
| Schizophrenia | 144 (1.1) | 196 (2.5) | 0.104 | < 0.001 |
| Questionnaire of special health checkups | | | | |
| Q1: ④ Not very good or ⑤ Not good | 1069 (8.3) | 918 (11.8) | 0.116 | < 0.001 |
| Q2: ③ Somewhat unsatisfied or ④Unsatisfied | 880 (6.9) | 756 (9.7) | 0.105 | < 0.001 |
| Q3: ① Yes | 12376 (96.1) | 7302 (93.0) | 0.135 | <0.001 |
| Q4: ① Yes | 3464 (26.9) | 2415 (30.8) | 0.086 | <0.001 |
| Q5: ① Yes | 2637 (20.5) | 1789 (22.8) | 0.056 | <0.001 |
| Q6: ① Yes | 1519 (11.8) | 1095 (13.9) | 0.064 | < 0.001 |
| Q7: ① Yes | 6920 (53.7) | 4805 (61.2) | 0.152 | < 0.001 |
| Q8: ① Yes | 2354 (18.3) | 1752 (22.3) | 0.101 | < 0.001 |
| Q9: ① Yes | 8071 (62.7) | 4326 (55.1) | 0.154 | < 0.001 |
| Q10: ① Yes | 1869 (14.5) | 1716 (21.9) | 0.191 | < 0.001 |
| Q11: ① Yes | 2818 (21.9) | 2316 (29.5) | 0.175 | < 0.001 |
| Q12: ① Yes | 471 (3.7) | 396 (5.0) | 0.068 | < 0.001 |
| Q13: ① Yes | 10847 (84.2) | 6269 (79.9) | 0.113 | < 0.001 |
| Q14: ① Yes | 12324 (95.7) | 7332 (93.4) | 0.100 | < 0.001 |
| Q15: ① Yes | 12388 (96.2) | 7482 (95.3) | 0.042 | 0.003 |
| SAGA score | | | | |
| Low–risk: 0–5 score | 11053 (86.4) | 6064 (77.2) | 0.243 | <0 .001 |
| Intermediate–risk: 6–11 score | 1694 (13.2) | 1701 (21.7) | | |
| High–risk: 12–18 score | 45 (0.4) | 85 (1.1) | | |

Abbreviations: y.o, years old; BMI, body mass index.
